# Supplementary material for: Are carbon emissions trading and green financial instruments synergistic? -Comprehensive quantitative research based on content analysis
Source: PLoS One. 2024 Mar 7;19(3):e0298601. doi: 10.1371/journal.pone.0298601 (PMC10919601; doi:10.1371/journal.pone.0298601)
Supplement: S1 Data — (ZIP) [file pone.0298601.s001.zip › Data/地级市Z_F工作报告.zip!/─┌├╔╣┼/║⌠║═║╞╠╪╩╨/└┤╫╘╣1⁄2╓┌║┼í╛┬φ┐╦╩2╛▌═°í┐_2013.docx]

各位代表：

　　现在，我代表市人民政府向大会报告工作，请予审议，并请各位政协委员和列席会议的同志们提出意见。

　　一、过去五年和2012年工作回顾

　　市十三届人大一次会议以来的五年，是我市改革开放和现代化建设取得显著成就的五年。五年来，我们紧紧围绕打造“两个一流首府”要求，坚持富民与强市并重的发展方针，全力推进“一核双圈一体化”发展战略，沉着应对前进中的各种风险和挑战，全市经济社会发展实现重大跨越，完成了本届政府确定的各项目标任务。

　　(一)综合经济实力大幅提升。预计地区生产总值由2007年的1128.7亿元增加到2460亿元，年均增长12.9%，人均地区生产总值超过84000元。地方财政总收入由120亿元增加到316亿元，年均增长21.4%。固定资产投资累计完成4650亿元，年均增长 16.1%。社会消费品零售总额由443.4亿元增加到1015亿元，年均增长18%。主要经济指标均实现翻番。三次产业比例由5.5：36.8：57.7演进为5.1：36.1：58.8。以奶牛规模化养殖、设施蔬菜种植为主的现代农牧业发展步伐加快，奶牛规模化养殖率由不足20%提高到70%，蔬菜保护地建成面积由1.2万亩增加到6.5万亩。现代工业体系建设取得新进展，乳业、电力、石化、冶金化工、生物发酵等优势特色产业得到进一步巩固和提升，光伏、电子信息、新材料等新兴产业加快发展，初步构建起多极支撑、多元发展的工业体系。全市工业增加值由2007年的346.2亿元增加到720亿元。商贸流通、餐饮住宿、交通运输等传统服务业全面提升，云计算、现代物流、金融保险、会展、旅游、中介等现代服务业快速发展，拉动增长、辐射周边、服务全区的能力明显增强。第三产业增加值由2007年的651.1亿元增加到1450亿元，年均增长13.5%。

　　(二)城市面貌发生显著变化。我们按照打造一流首府城市要求，加快推进城市“一街五区”新区建设，在老城区实施“新亮美绿净畅”工程，城市建设实现“六个明显提升”。一是中心城区的辐射带动力明显提升。建成区面积由2007年的150平方公里扩展到230平方公里，市区常住人口由140万人增加到220万人。二是城市基础设施承载能力明显提升。对城市主次干道连同地下管网进行大范围改造建设，五年累计改造道路41条，新建续建道路33条、桥梁通道27座，形成“三线环城、八横八纵”道路网。城市生活污水集中处理率和生活垃圾无害化处理率分别达到96%、98%，再生水利用率超过30%，集中供热普及率超过80%，二环路以内99%的居民用上天然气。新增加公交车辆930台，新开通和延伸公交营运线路69条，基本覆盖了城区全部主次干道。三是城市综合服务功能明显提升。建成一批城市商业综合体和星级酒店，新建铁路东客站等一批大型公用项目，城市公共服务设施不断完善。四是城市建设的品位与特色明显提升。对草原文化、历史文脉、民族特色进行挖掘和塑造，修缮保护了一批文化古迹，改造建设了一批特色街区。完成全长56公里的环城水系及景观建设，新建敕勒川公园、成吉思汗公园等一批城市园林绿地，建成区绿地面积增加1100公顷，人均公共绿地由13.9平方米提高到16.4平方米。五是群众居住环境明显提升。对全市1123个老旧小区、292条小街巷进行了改造，有效改善了群众居住和出行条件。切实加大环境保护工作力度，市区空气质量优良天数由331天增加到347天，通过了国家环保模范城市验收。六是城市管理水平明显提升。全面加大拆违拆临力度，加强市容环境管理和市政设施维护，着力提高城市精细化管理水平，城市面貌不断发生新变化。

　　(三)城乡基础设施进一步完善。我们按照城乡一体、区域一体的发展思路，加快推进小城镇建设，先后在五个旗县召开两个文明建设经验交流暨城镇建设现场会，有力促进了各旗县政府所在地及重点建制镇建设。连接全市城乡的呼武公路复线、209国道和林至清水河段一级路、金盛路等重点公路全线贯通，乡通油路、村通公路全面实现，“一核双圈一体化”的公路网基本形成。全市公路通车里程增加711公里，路网密度比2007年提高11.7%。加强水利建设，启动实施引黄入呼二期工程，完成20座中小型病险水库除险加固，对8处万亩以上灌区进行了节水改造，新增有效灌溉面积44万亩。大力实施农村饮水安全工程，解决34.8万人饮水安全问题。实施110千伏及以上输变电项目44项，城乡电网结构进一步完善。启动大青山生态综合治理保护工程，大力推进退耕还林、天然林保护等重点生态建设工程，五年累计完成林业生态建设321万亩，全市森林覆被率由20.7%提高到29.9%，在西北省会中第一个建成国家森林城市。

　　(四)改革开放深入推进。我们始终坚持把深化改革开放作为推动发展的动力，积极推进重点领域改革。农村综合改革不断深化，集体林权制度改革全部完成。医改成果普惠城乡居民，全市政府办基层医疗机构基本药物实行零差率销售，药价平均降幅30%。文化体制改革逐步深化，市电台和电视台合并，呼和浩特民族演艺集团成立。政府机构改革和事业单位绩效工资制度改革积极推进。集体企业改革、财税管理体制改革取得新进展。推进地方金融改革，小额贷款公司和融资性担保公司得到快速发展。制定出台促进民营经济加快发展50条意见，鼓励支持和引导非公有制经济发展的力度全面加大，个体工商户、私营企业户数分别达到12.2万户和3万户。对内对外开放进一步扩大，五年累计引进国内资金2156亿元，年均增长13.5%;实际利用外资38亿美元，年均增长10.1%;实现外贸进出口总额70亿美元, 年均增长25.3%。

　　(五)人民生活水平显著提高。我们始终坚持以人为本，不断加大保障和改善民生工作力度。城镇居民人均可支配收入由2007年的16920元提高到2012年的32350元，年均增长13.8%;农民人均纯收入由6121元提高到2012年的11240元，年均增长12.9%。就业工作不断加强，全市累计新增就业18.1万人，实现零就业家庭至少1人就业。社会保障体系建设成效显著，城乡基本养老保险制度全面建立，全民医保基本实现。连续五年提高企业退休人员养老金，先后三次提高失业金标准。城镇职工和居民基本医疗保险最高支付限额分别达到31万元和21万元，住院费实际平均报销比例提高到77%和55%。新型农村合作医疗实行市级统筹，政策范围内住院费用报销比例提高到75%，并建立大病补偿机制。城乡低保水平、五保供养、孤儿供养、城镇“三无”人员供养标准达到西部省区领先水平。实施城乡医疗、教育、取暖等专项救助，累计受益群众70多万人次。扶贫工作深入开展，贫困人口收入较快增长。开工建设各类保障性住房11.6万余套，基本解决了市区人均住房面积不足13平方米的低保家庭住房困难问题。稳步推进农村危旧房改造工作，5200多户农村困难群众居住条件得到改善。

　　(六)社会事业全面进步。我们始终坚持统筹兼顾，高度重视社会事业发展，着力健全社会公共服务体系。坚持教育优先发展，义务教育、高中教育以及蒙语授课的幼儿园全部实现免费。校安工程及中小学标准化建设工程扎实推进，全市新增校舍93万平方米，新增教学班623个。职业教育、民办教育、民族教育得到稳步发展。全市各级各类教育质量不断提升。医疗卫生事业得到快速发展，全市医疗卫生机构达到2163个，为全市166万城乡居民建立规范化电子健康档案。国家创新型城市试点工作全面展开，五年组织实施重大科技专项224项，有39项科技创新成果获得自治区科技进步奖，专利申请量和授权量均居自治区首位。奶牛性控技术、粉煤灰综合利用、通信塑料光纤等一批自主创新成果实现产业化。公共文化服务体系建设不断加强，基本实现乡乡有标准文化站，31家博物馆、纪念馆等公共文化服务设施向公众免费开放。文化产业和文艺创作繁荣发展，昭君文化节先后获得“全国十大品牌节庆”、“优秀民族节庆”等称号，一批优秀文艺作品走向全国。新闻出版、广播电视事业进一步发展。体育设施得到完善，群众性体育活动广泛开展，竞技体育屡创佳绩。在自治区第十二届运动会上，我市代表团蝉联金牌总数第一。人口素质不断提高，人口出生率控制在9.09%。。深入推进社会矛盾排查化解，一批信访突出问题得到妥善解决。坚持严厉打击各类刑事犯罪，不断强化社会治安综合治理，平安首府建设扎实推进。安全生产形势保持平稳，食品药品监督管理工作进一步加强。社区建设深入推进，全市近80%的社区用房达到300平方米以上。民族宗教工作取得新的成绩，平等团结互助和谐的社会主义民族关系得到进一步巩固和发展。深入开展拥军优属、拥政爱民活动，国防和民兵后备力量建设不断加强，连续七次被国家命名为“双拥模范城”。重视工会、共青团、妇联等人民团体工作，支持残疾人、妇女儿童和老龄事业发展。人防、地震、防汛、消防、气象、红十字会、关心下一代等各项事业都取得新成绩。

　　(七)政府自身建设不断加强。自觉接受人大依法监督和政协民主监督，主动向人大及其常委会报告工作，向政协常委会通报情况，累计办理市人大代表议案、建议、意见938件，市政协委员提案2783件。认真听取各民主党派、工商联和无党派人士意见，决策科学化、民主化程度不断提高。依法治市工作取得新成效，“五五”普法全面完成，“六五”普法顺利实施。在全市持续深入开展“转变工作作风、强化服务意识、打造一流首府机关形象”、“首府服务提升年”等专项活动，取得明显成效。全力推进三级公共服务体系建设，大力改革行政审批制度，市直部门行政审批项目由708项减少到233项，取消和停止征收95项行政事业性收费项目，行政审批事项实现100%集中受理，审批过程100%电子监控。严格执行党风廉政建设责任制，加大源头防腐、纠风治乱力度，加强对政府投资项目的管理和审计，严肃查处了一批违法违纪案件。

　　刚刚过去的2012年，面对较为困难的经济形势，我们既立足当前，牢牢抓住项目建设、民生改善和社会管理创新等工作重点，全面落实稳增长、调结构、促和谐的各项措施，又着眼长远，全面谋划首府未来发展，着力优化城市建设及产业发展布局，着手抓了一系列打基础、利长远的大事实事。全市国民经济继续保持平稳较快发展势头，并呈现出结构优化、层次提升、后劲增强的积极变化，社会各项事业取得新成绩。一是项目建设取得重大突破。建立了强力高效的项目推进机制，在经济社会发展各领域实施一批重大项目，全市组织实施亿元以上项目370项，其中10亿元以上项目76项。完成全社会固定资产投资1300亿元，增幅达26%，有效扭转近年来项目建设乏力、投资增速下滑的局面。二是工业经济企稳回升。在宏观经济下行和我市部分骨干企业停产扩能改造的双重压力下，通过狠抓项目建设和政策扶持，全年新增规模以上企业20家，是近年来新增数量最多的一年。一批大型骨干企业在逆境中得到进一步发展壮大。中石油500万吨炼油项目正式投产，TCL、创维两大液晶电视项目顺利建成，有效增强了对全市工业发展的支撑力。理顺了国家级经济技术开发区的管理体制，启动了总规划面积100平方公里的沙尔沁工业新区建设。三是农村重点工作迅速推进。全年新开工建设蔬菜保护地7万亩，建成4万亩，年内新建成保护地是历年蔬菜保护地面积的1.6倍。建成标准化奶牛养殖牧场15个，新增奶牛8万头。大力推进休闲观光试点村的规划与建设，部分项目建设已取得实质性进展。扎实推进新一轮扶贫开发，确定并实施了30个整村推进扶贫开发项目，取得阶段性成效。四是服务业继续保持较快发展势头。大力推进以云计算为代表的高端服务业发展，中国电信项目主体工程、中国联通数据机房楼完成封顶，中国移动数据中心已完成全球招标和规划设计工作。高度重视现代物流、楼宇经济发展，加快总部基地建设，实施和建成一批城市综合体项目。大力发展旅游业，规划启动了大青山生态综合保护、哈素海文化旅游区、托县黄河湿地、清水河老牛湾国家地质公园等大型生态旅游项目，不断改善提升旅游环境，预计全年旅游接待人数达到1845万人次，实现收入267亿元。金融业加快发展，新引进7家金融机构，又有华蒙金河、蒙草抗旱2家企业成功上市。会展业发展迅速，以中国民族商品交易会为代表的一大批生产型、生活型展会取得明显成果。五是城市改造建设取得新突破。下大力组织实施了一批主次干道、地下管网、小街巷、老旧小区改造工程，特别是地下管网改造是历年来力度最大的一年。全年完成核心区道路改造15条、小街巷59条，改造老旧小区325个。对42个城中村进行改造，动迁面积超过1000万平方米。大力实施城市分质分类配水计划，初步实现了对重点工业用水企业分类供水目标。启动白塔机场迁建的论证工作，前期工作取得积极进展。完成了城市轨道交通线网规划。继续提升城市管理水平，基本完成智能化交通管理和视频监控系统建设。六是以改善民生为重点的社会建设得到加强。不断加大民生领域的投入，加强公共就业和人才服务体系以及社会保障体系建设，各类保障对象的收入水平进一步提高。加快教育、医疗等各项社会事业发展，市蒙中医院、妇幼保健院等一批项目全面开工建设。在试点基础上全面推开社区网格化管理，初步构筑起符合首府实际的社区网格化服务管理体系和运行机制。加强应急管理体系建设，有效应对和处置突发事件，全力组织实施抗洪抢险工作。成功完成一系列重大维稳任务，集中解决了一批企业转制、城镇动迁、土地征用等历史遗留问题，营造了良好的社会环境。七是各项重点改革取得新的进展。全市公共资源交易管理体制改革顺利推进，政府采购、工程交易、土地拍卖统一纳入市公共资源交易中心实施交易，建立了统一、公开的资源交易平台。财政管理体制改革继续深化，投资管理以及教育、医疗、文化等各领域改革进一步向前推进。

　　需要说明的是：根据全国、全区整体经济形势的变化和国家、自治区对经济指标的调整情况，综合考虑我市前三季度部分骨干工业企业停产扩能改造、环保设施改造等因素带来的影响，进入第四季度，我们对2012年年初确定的部分主要国民经济年度计划指标进行了调整，并报经市十三届人大常委会第37次会议予以批准。

　　各位代表，过去五年我们取得的成就，凝聚着全市人民的辛勤劳动，体现着社会各界的贡献付出。在此，我代表市人民政府，向全市各族干部群众，向人大代表、政协委员，向各民主党派、工商联、人民团体、社会各界，向中央和自治区驻呼单位，向驻呼解放军和武警官兵，向所有关心支持呼和浩特建设发展的同志们、朋友们，表示崇高的敬意和衷心的感谢!

　　在肯定成绩的同时，我们也清醒认识到，我市经济社会发展中还存在不少问题。一是整体经济实力还不够强，工业短板尚未补齐，现代服务业发展不够充分，现代农业建设仍处于起步阶段。二是城市建设水准、服务功能、畅通能力与一流首府城市的要求还有不小差距，存在许多不配套、不完善、精细化程度不够的问题。三是城乡发展不协调的问题还没有得到根本解决，农村基础设施、公共服务、社会管理各方面欠账较多，推进城乡一体化发展的任务十分艰巨。四是社会建设和管理存在薄弱环节，保障和改善民生、创新社会管理的任务仍然繁重，部分群众生活还比较困难。五是政府自身建设有待进一步加强，基层群众对机关作风、发展环境还有许多不满意的地方。我们一定要以对人民高度负责的精神，加快解决这些问题。

　　二、2013年工作部署

　　2013年是全面深入贯彻落实党的十八大精神的开局之年，是实施“十二五”规划承上启下的关键一年，也是新一届政府任期的起步之年，做好今年的各项工作意义重大。纵观发展大势，尽管我们所处的发展环境复杂多变，经济下行的压力仍然很大，我市在转型发展中面临诸多矛盾和挑战，但总的看，我市仍处于机遇大于挑战、可以大有作为的战略机遇期。我们既有来自中央与自治区的有力政策支持，也有首府独特的区位优势、人才优势、发展要素集聚优势。

　　更为重要的是，我市正处于工业化、城镇化、信息化和农牧业现代化加速推进期，建设空间广阔，投资需求巨大，发展动力充足。

　　2013年政府工作的总体要求是：认真贯彻党的十八大以及自治区党委九届四次、五次全委会和市委十一届三次全委会精神，以科学发展观统揽全局，坚持富民与强市并重，按照打造“两个一流”、建设“活力首府、美丽首府、和谐首府”、实现“两个率先”要求，着力优化城乡经济社会发展布局，牢牢抓住项目建设这个推动发展的重要抓手不动摇，把促增长、转方式、调结构、惠民生更好地结合起来，为实现在全区率先全面建成小康社会及城乡一体化迈出坚实的步伐。

　　2013年经济社会发展主要预期目标是：地区生产总值同比增长12%;规模以上工业增加值增长20%;地方财政总收入增长12%;固定资产投资增长15%;社会消费品零售总额增长14%;城镇居民人均可支配收入和农民人均纯收入均增长12%;城镇登记失业率控制在3.8%以内;居民消费价格指数涨幅控制在3.5%左右;节能减排指标完成自治区下达任务。

　　今年要重点抓好十个方面工作：

　　(一)继续大力推动工业经济加快发展，增强工业经济支撑带动力

　　我们要进一步优化工业布局，狠抓项目建设，做大工业总量，推动产业升级，切实增强工业经济的整体发展水平。

　　按照分别打造千亿元级工业集中区的目标，培育支柱产业，加大招商引资力度，加快推进工业园区建设。抓好沙尔沁工业新区的规划编制及基础设施建设，建设以新能源和新材料、装备制造业为主的我市未来工业核心区;推进托克托工业园区与清水河工业园区联合发展，建设以电力、重化工业、新型冶金、生物制药为主的产业基地;提升盛乐经济开发区整体水平，重点打造以绿色食品加工、云计算为主的产业基地;合理规划扩大金桥开发区，延伸拉长产业链，建设以石化、光伏、航天科技军民融合产品转化等为主的产业基地;加快金山、鸿盛国家级高新技术产业开发区的整合升级工作，重点发展乳制品、电子科技、生命科学、装备制造及国家级研发、检测中心等高新技术产业。努力把以上几大工业集中区打造为优势突出、特色鲜明、基础完善、业态先进的现代化工业园区。

　　着力推进项目建设，全年工业固定资产投资同比增长20%以上。乳品加工业要全力支持伊利、蒙牛扩产提效，进一步延伸产业链，加大新产品的研发力度，发展高端乳制品。依托中粮集团，加快打造中粮食品产业园，推进食品产业集群化发展。电力产业要加快北方联合电力和林电厂、京能盛乐热电厂、大青山抽水蓄能电站建设进度，全力促进托电五期开工建设，积极谋划重点用电企业自备电厂、沙尔沁新区电厂等新项目建设。石化产业要在全力推动中石油500万吨炼油项目达产达效、呼包鄂成品油管道工程的同时，继续推进第二个500万吨炼油项目的各项前期工作。加大北控20亿立方米煤制气、斯莱登煤制烯烃等项目推进力度，促进天野化工煤代气、35万吨合成氨、60万吨大颗粒尿素项目早日开工。冶金产业要在加快推进大唐高铝粉煤灰三期、蒙西粉煤灰综合利用、广银铝业、光太铝业、东亚铝业等项目建设的基础上，继续大力引进铝深加工企业，全面提升冶金产业发展水平。生物医药产业要积极推进华蒙金河高效金霉素、盐霉素扩建、航天科技辅酶Q5新产品、健隆生化黄原胶扩产等项目建设，推动产业向高端化发展。电子信息产业要围绕TCL和创维两个液晶电视生产项目，加快上游配套企业引进步伐，推动电子信息产业集群化发展。光伏产业要着力抓好天津中环三期、华夏聚光吉瓦光伏中心、神舟硅业三期等项目建设，加快构建完整产业链。要大力发展新材料、装备制造业，加快推进浩源碳纤维、金三角塑料光纤、F-12高强度有机纤维等项目达产达效，积极促进航天科工集团高端装备制造及新材料产业园建设，着力提升产业发展层次。

　　加快中小企业创业基地建设，鼓励中小企业围绕大企业上下游产业链提供协作配套,建立稳定的产供销和技术开发等协作关系，推进产业集群化发展。全面落实促进民营经济发展的各项政策措施，激发民营经济创业活力。充分发挥工商联在发展民营经济中的桥梁助手作用。设立中小企业发展专项资金，在信贷融资、税费减免、项目审批、技术创新、节能减排、建设用地、人才培训等方面给予更大力度的倾斜和支持。年内重点扶持20户中小企业成长为规模以上企业。继续实施大企业扶持计划，通过3至5年的努力，全市销售收入超100亿元企业力争达到20家。

　　加强与国家、自治区有关部门的对接，争取更多的政策、项目和资金支持。强化工业经济运行监控，加大对企业服务协调力度，努力营造有利于企业发展的良好环境。完善信用担保体系，积极协调金融机构增加信贷投放规模，增强对重大项目和实体经济的支持力度。

　　(二)全面提升服务业水平，提高服务业对发展的贡献率

　　要进一步发挥首府区位优势以及人流、物流、信息流集聚优势，着力完善服务体系，增强综合服务功能，提升服务业发展水平。

　　全面推进云计算产业发展，推动中国电信内蒙古信息园、中国联通西北基地一期工程建成投产，中国移动数据中心完成年度建设任务，启动建设宽带资本云计算创业园和国家云计算研发中心，加快构筑服务全区、面向全国的云计算服务平台。着力引进关联度较高、成长性较好的配套项目，尽快培育形成集设备制造、软件开发、商务服务、人才培养为一体的云计算产业发展链条。

　　加快发展现代服务业。全力推进如意总部基地建设，制定出台优惠政策，争取年内再引进一批新的大中型企业总部。继续抓好银行、保险、证券等金融机构引进工作，支持内蒙古银行发展壮大，支持内蒙古金融投资集团牵头筹建自治区法人主体农牧业保险公司，推动金谷农合行转制发展。重视现代物流发展，抓好内蒙古煤炭电子信息交易服务中心和重点煤炭物流园区建设。加快推进大青山、哈素海、托县黄河湿地等重点旅游景区建设，力促老牛湾国家地质公园申报成功，认真做好国家A级景区创建工作，推动全市旅游业整体发展再上新台阶。全面完成内蒙古国际会展中心改造，精心组织第七届中国民族商品交易会等特色会展活动。

　　立足于满足城乡居民消费结构升级的需求，改造提升传统服务业。加快万达广场二期、海亮广场二期、大商城、金宇新天地等城市综合体项目建设，推动城市由单中心、高集聚向多中心、多组团发展。继续加大商业街区开发建设力度，提升大盛魁、大召区块等特色街区的集聚能力，推动城市各类商业网点合理布局，方便群众消费。围绕满足社区居民需求，继续大力发展社区服务业，加快建立高效便捷的社区服务体系。认真落实国家促进消费政策，拓展消费服务领域，增强消费对经济的拉动作用。

　　加快首府地区航空业发展，支持各航空公司在我市建立分支机构，加大新航线的培育力度，实现“畅行区内、通达省会、首府中转、开拓国际”的目标。

　　(三)毫不松懈地抓好高效特色农牧业，推进城乡一体化发展

　　继续加大农业投入，支持和引导多种经营主体参与现代农牧业建设，推动农业增效、农村发展、农民增收。扎实推进“菜篮子”工程建设，年内新开工蔬菜保护地5万亩，完成7个标准化蔬菜育苗中心建设;抓好奶牛规模化牧场建设，年内新建千头以上奶牛牧场12个，新增奶牛10万头，规模化养殖水平提高到80%;启动实施“优质牧草种植行动”计划，高标准建设15万亩苜蓿草生产基地。加快发展“农家乐”、特色种养业，建成一批集休闲娱乐、餐饮住宿、观光旅游功能为一体的特色村落，推动农牧业与服务业融合发展。同时，要继续因地制宜加大对肉羊、马铃薯等产业的支持扶持力度，促进农民多渠道增收。

　　着力完善农牧业社会化服务体系。加强基层技术推广机构建设，提升基层农技服务水平。创新良种良法推广应用手段，突出农业科技园区示范带动功能，鼓励有实力的个人、企业、集体组织兴办农技服务实体机构。加快农业专业合作社建设步伐，提高农牧业生产经营的组织化程度。加大农业生产实用技术培训力度，着力培养懂生产、会经营、善管理的新型农民。与自治区相关部门共同推进呼和浩特乳品研发和检测中心的改造提升，强化农畜产品安全监管。

　　按照在全区率先实现城乡发展一体化的目标，坚持分类指导、梯次推进，加快推进新农村建设。首先加快旗县城关镇的建设，使小城镇成为连接城市、辐射农村的重要公共服务平台和新的区域经济增长点。今年市政府要专门制定出台小城镇建设支持政策，在资金投入、基础设施建设、现代产业建设、公共服务体系建设等方面加大支持力度，努力构建城乡一体化的重要节点。其次分层次制定乡村发展建设规划。一是继续将城中村、城边村纳入城市整体发展规划，加大动迁改造力度，实现城乡一体化，居住、就业、公共服务均等化。二是对农业生产及发展其他产业条件较好的村，加大新农村建设力度。今年要编制完成150个村的新农村建设发展规划，年内完成20个以上新农村示范村的建设。三是对地处偏远、交通和生态条件较差的村，以减少贫困人口为目标，继续采取移民扶贫、异地产业扶贫、教育扶贫等综合措施，提升扶贫开发工作水平。

　　(四)优化城市空间发展布局，加快打造新的经济增长极

　　我们要立足首府长远发展，对城市发展整体布局进行科学调整，着力打造“四个100平方公里”功能区，为首府经济快速增长提供新引擎。除国家级经济技术开发区沙尔沁100平方公里工业新区外，今年还要加快以下三个区域的规划和建设：

　　一是全面启动白塔机场迁建前期工作，同步推进机场搬迁后城市东部100平方公里新区规划工作，高水平、高档次对这一区域进行统一规划设计，彰显特色、体现人文、完善功能，努力将该区域建设成为代表首府城市形象和发展水平的特色新区。

　　二是依托新机场建设和铁路、公路交通网，在新机场周边规划100平方公里空港产业区，发展保税物流、第三方物流、先进制造业等空港经济，打造航空经济产业链，为现代产业发展提供新平台和有力保障。

　　三是全面实施大青山南坡150平方公里生态保护综合治理工程整体规划，加快推进工程建设。以保护水源、恢复生态为重点，加大造林绿化力度，筑牢首府生态屏障。结合生态治理和新农村建设，进一步完善大青山南坡休闲观光功能，抓好区域内村庄的改造搬迁和水电路等基础设施建设。加快乌素图沟、小井沟等地生态保护及精品景观带和景观节点建设，全力抓好赛马场搬迁、万亩草场等一系列休闲观光项目的建设，努力把大青山建成首府城市后花园。

　　(五)继续加大中心城市改造建设力度，进一步提升首府城市建设水平

　　坚持把推进城市建设与改善民生紧密结合起来，在完善功能、提升品位和精细化管理上下功夫。

　　加快城市基础设施建设。进一步优化路网布局，加大城市新区与核心区的连通力度，新建道路、桥梁及人行通道47条(座)。按照先地下、后地上的原则，稳步推进核心区道路及地下管网改造建设工程，全年改造核心区道路12条、小街巷55条。实施“大公交”发展战略，全年新增公交车400台，加快公交场站建设，免费乘坐公交车的老年人范围扩大到60岁以上。完成城市轨道交通建设规划编制，推动轨道交通的建设。完成火车站东站南广场建设，规划建设北广场。全面推进城市分质分类配水目标，加快实施中水回用工程，扩大中水利用率，提高城市供水安全和质量。加强供热保障能力建设，新建、扩建热源厂4座，建设热力站34座，新增供热能力900万平方米以上。继续加大便民基础设施建设力度，新建便民市场100处以上。加强环卫设施建设，完成城市垃圾无害化处理场扩建工程和旧场封场工作，在市区新建4座大型垃圾中转站、20座压缩式垃圾转运站和50座环保节能公共厕所。

　　加大街景改造和城市绿化力度。启动成吉思汗东街街景改造工程，打造蒙元文化特色街区。深入推进国家园林城市创建工作，扎实开展新一轮美化、绿化，完善环城水系景观带，完成大黑河赛罕段综合治理和景观工程，启动哈拉沁沟地质公园、驿林公园等大型公园建设，全年新建、续建、改造公园和游园9个，新建绿地13块，实施一批道路绿化、景观节点改造工程。

　　全力抓好老旧小区改造和保障性住房建设。将老旧小区整治纳入住房保障体系，在资金投入和政策扶持上给予重点保障，确保老旧小区在功能设施、美化绿化、物业管理等方面一步改造到位，创建更加优美整洁的社区环境。今年计划改造老旧小区300个以上。配合老旧小区改造，完成200万平方米既有居住建筑节能改造。加强保障性住房建设，年内新建公租房5000套，经济适用住房1000套。加大城中村改造力度，年内完成动迁面积1000万平方米以上。继续将部分城中村改造纳入城市棚户区改造工作中，改造城市棚户区1万户，开工建设回迁房80万平方米。

　　着力提升城市科学化管理水平。全面完成数字化城市管理系统建设，充分发挥智能化交通管理和视频监控系统作用，提高交通组织管理水平，增强道路通行能力。严格执行城市建设规划，保证规划刚性落实。加强对城乡结合部和拆迁改造区域的综合整治，坚决制止和依法打击私搭乱建等违法行为。落实“门前三包”责任制，加强市政和园林绿化设施的维护，增设一批环卫设施，继续适当提高环卫工人待遇，积极开展户外广告、流动摊点、建筑工地、公共场所、小街小巷等重点区域卫生环境专项治理行动。重视解决城市防洪、内涝、冰雪等严重影响群众日常生活和财产损失等问题，提高应对各类突发事件和公共安全保障能力。

　　(六)着力提升文化软实力，推进文化大发展大繁荣

　　充分发挥文化在引领风尚、服务社会、推动发展等方面的重要作用，继续深入推进文化体制改革，着力培育特色鲜明的文化产业，全面构建服务广泛的公共文化服务体系，加快建设民族文化大市。

　　精心组织、深入开展全国文明城市创建工作，全面提升城市文明程度和市民文明素质。加强社会公德、职业道德、家庭美德、个人品德教育，倡导开放包容、诚信友善，培育自尊自信、理性平和、积极向上的社会心态。推进未成年人思想道德建设，为未成年人健康成长营造良好文化环境。强化社会主义核心价值体系学习教育，用社会主义核心价值体系引领社会思潮、凝聚社会共识。

　　加快建立完善的公共文化服务体系。加强城乡公共文化基础设施建设，完成市民族文化演艺中心、群众艺术馆建设工程。推进重点文化惠民工程，坚持和完善公共文化服务设施向社会免费开放政策，支持民间博物馆建设并逐步纳入政府补贴范围。年内新建区域服务中心文化站10个，农家书屋290家，免费送戏下乡100场，农村电影放映12000场以上。广泛开展群众性文化活动，办好昭君文化节、少数民族文化旅游艺术节。加大对历史文化街区、历史建筑、优秀民间艺术的保护力度，完成原内蒙古博物馆、将军衙署、乌素图召修缮保护工程，启动丰州古城遗址公园建设，提升历史文化名城内涵。

　　全力推动文化产业做大做强。围绕新闻出版、影视动漫、文艺创作等领域，培育扶持一批优秀企业发展壮大。支持好市电视台和广播电台合并后及市新组建的演艺集团的发展，深化体制机制改革、鼓励创新，促进文化与科技融合，发展新型文化业态，提高各类文化产业规模化、集约化、专业化水平。充分利用首府地区文艺院团集中的优势，认真抓好文艺精品创作，推出一批展现蒙元文化、地区文化的精品文化工程，增强我市文化对外影响力。加快文化产业集聚区建设，推动呼和浩特文化产业园、大盛魁文化创意产业园等重点园区做大做强。

　　(七)继续加强以改善民生为重点的社会建设，促进社会和谐

　　坚持富民优先，加大新增财力倾斜力度，积极推进社会建设，让全市人民更多地分享改革发展成果。

　　抓好促进就业工作。实施更加积极的就业政策，完善就业服务体系，拓宽就业渠道，开发就业岗位，做好以高校毕业生为重点的青年就业工作和农村转移劳动力、城镇困难人员就业工作。大力扶持劳动密集型产业、中小型和微型民营企业发展，提高就业吸纳能力。加快创业实训基地、孵化基地和创业园建设，促进创业带动就业，重点扶持高校毕业生和农民工返乡创业。加强人力资源市场建设，强化职业技能培训，提升劳动者就业能力，增强就业稳定性。着力维护劳动者合法权益，构建和谐劳动关系。全年新增城镇就业4万人。积极开发公益性岗位，今年市四区和旗县公益性岗位补贴每月分别达到1200元和1100元。

　　大力推进城乡社会保障体系建设。切实解决未参保集体企业、厂办大集体职工参加城镇企业职工基本养老保险问题，进一步扩大社会养老保险覆盖面，年内实现城乡居民养老保险制度全覆盖。制定出台原国有企业大龄困难下岗失业人员社会保险缴费救助政策，解决好大龄下岗失业人员社会保险金“断保”问题。进一步落实职工基本医疗保险市级统筹政策。将普通门诊统筹与慢性病并轨，提高城镇职工门诊统筹待遇水平。完善社会救助体系，加强对城乡低收入困难群体的救助、帮扶工作。严格落实城乡居民最低生活保障和农村五保供养保障政策，足额发放高龄老人津贴。加快发展养老福利事业，完善适度普惠型的老年福利和养老服务体系。积极引进、培育和发展居家养老服务组织，为老年人提供高效便捷服务。结合扶贫开发和完善农村最低生活保障制度，在农村积极推进互助养老幸福院建设。鼓励和支持慈善事业发展壮大。

　　围绕“办人民满意教育”目标，大力支持教育优先发展，巩固和提升我市在自治区的教育强市地位。深入实施学前教育三年行动计划，设立专项资金，支持新建和改扩建幼儿园，年内完成10所改扩建任务。继续推进呼市二中西校区、十七中等中小学校标准化建设。强化对中小学、幼儿园安全防范和校车安全管理。加强职业院校实训基地建设，大力扶持与社会需求紧密对接的高质量职业院校。积极推进素质教育，促进学生德智体美全面发展。高度重视民族教育，继续鼓励和规范民办教育发展，支持高等教育发展。

　　继续深化医疗卫生体制改革，加快推进医疗卫生服务设施建设，抓好市妇幼保健院、蒙中医院、结核病防治所、第一医院老年病房楼建设。巩固基本药物制度实施成果，落实好基层医疗卫生机构基本药物的配备使用及医保支付政策。稳步推进县级公立医院改革试点工作，积极发展蒙医中医事业，加强基层医疗卫生服务人才培养。切实抓好人口和计划生育工作，提高出生人口素质，完成市人口计生服务中心建设。全面实施残疾人“五项工程”，筹建市级康复中心大厦，推动残疾人事业加快发展。切实发挥工会、共青团、妇联等群团组织的桥梁纽带作用，继续做好妇女儿童、老年人、红十字会工作。大力发展体育事业，加快体育基础设施建设，抓好调整规划后的市体育运动学校和市游泳馆项目建设，构建多元化全民健身服务体系。

　　积极开展社会管理创新综合试点，继续推进社区网格化服务管理，年内新增社区协警450人，加快社区综治和服务平台建设，充分发挥社区在服务人民群众、参与社会管理、化解社会矛盾等方面的作用。全面推进社区标准化建设，今年全市社区用房力争全部达到300平方米以上，市四区各建1个5000平方米以上的精品示范社区。办公、活动场所在300平方米以上的社区力争成立居家养老服务中心或老年活动中心。围绕打造“一刻钟服务圈”，切实抓好社区便民市场、社区卫生服务中心和社区文化娱乐中心建设，不断满足市民日常生活、就近医疗和休闲娱乐的需求。落实信访工作领导责任制，深入开展领导干部大接访、大走访活动。进一步完善社会稳定风险评估、社会舆情分析、群众利益诉求表达等工作机制，更好地保障和维护群众利益。加强社会治安综合治理，实现人防、物防与技防有效结合，形成全方位、立体化、全天候的社会治安防控体系，依法严厉打击各类违法犯罪行为，提升平安首府建设水平。健全和落实各项安全生产制度，抓好工业生产、交通运输、建筑安装、消防等重点领域的隐患排查治理，坚决防止重特大安全事故发生。健全完善防灾减灾和应急管理体系。全面加强食品药品安全责任体系建设，完善监管手段，提升监管能力，深入开展专项整治，建立健全食品药品监管的长效工作机制。加快“放心粮油工程”建设，建立成品粮应急保障体系。全面贯彻党的民族宗教政策，依法管理民族宗教事务。坚持走军民融合式发展的路子，强化全民国防教育，加强国防动员和后备力量建设，深化应急力量建设成果，做好拥军优属、拥政爱民工作，不断巩固新形势下的军政军民团结。扎实做好统计、档案、气象、地震、外事侨务、科协等工作，推进各项社会事业全面发展。

　　(八)加强生态环境和基础设施建设，夯实可持续发展基础

　　全力抓好资源节约和环境保护工作。严格落实耕地保护目标责任制，加强矿产资源保护和管理，有效降低土地、能源消耗强度。落实最严格的水资源管理制度，积极创建节水型城市。全面抓好工业、建筑、交通运输、公共机构等领域节能，加快淘汰落后产能。抓好节能与新能源汽车的示范推广，推进节能环保新技术、新产品应用。积极发展低碳经济、循环经济，提高资源综合利用水平。实行严格的环境保护制度和准入标准，控制高能耗和产能过剩企业扩大产能，防止污染项目向我市转移。大力推进燃煤电厂脱硫、脱硝工程，强化水、大气、噪声、土壤等污染防治和农村面源污染防治。加快金桥开发区污水处理厂建设，确保旗县所在地污水处理厂正常运行。全面完成工业园区废水集中处理设施建设，实现重点工业企业排污许可证全覆盖。巩固国家环保模范城市创建成果，开展PM2.5监测，不断改善环境质量。

　　继续实施天然林保护、三北防护林、退耕还林等重点生态工程，完成封山育林20万亩，人工造林13万亩。加强蛮汉山、黄河流域、低山丘陵等地区生态治理和重要水源地涵养林保护工程建设，全面完成大青山南坡绿化工程主体建设任务。启动京藏高速、绕城高速、城壕高速等通道绿化工程，切实抓好城区、村庄绿化和农田防护林建设工作。加强生态文明宣传教育，在全社会形成共同爱护生态环境、共同享受文明生态的良好风气。

　　加快G7高速、呼杀高速等出区通道重点公路续建项目建设，新开工武川至葛根塔拉、国道110线呼和浩特至协力气段等一级公路，推进行政村通油路、水泥路，加快汽车客运东枢纽站建设进度。推进引黄入呼二期扩建工程，实施好病险水库除险加固和节水灌溉工程，加快中小河流治理进程，切实提高用水保障能力。继续加强农田基本建设及水土保持工作，深入推进农村饮水安全工程。新建续建110千伏及以上输变电工程31项，加快电网建设步伐。

　　(九)深入推进改革开放和科技创新，建设创新型城市

　　要进一步深化改革、扩大开放，着力加强科技创新体系建设，加快建设创新型城市，为首府经济社会发展增添新的动力。

　　积极推进重点领域、关键环节改革。加快完善促进城乡发展一体化体制机制，促进城乡要素平等交换和公共资源均衡配置。深化收入分配改革，进一步规范公务员津补贴，推进事业单位绩效工资制度改革，完善企业职工工资正常增长和支付保障机制。继续推进三级公共服务平台标准化建设，深化集中审批服务，抓好政务公开工作，推动政府职能向创造良好发展环境、提供优质公共服务、维护社会公平正义转变。加快全市公共资源交易中心建设，推进中心有效运行，进一步完善公共资源交易监管体制和机制，实现公共资源“阳光交易”全覆盖。稳步推进财税体制改革，深化预算制度改革，完善公共财政体系，强化国有资产监管。支持旗县区之间打破行政体制制约，突破空间地域限制，合作共建产业园区，形成资源共享、优势互补的发展格局。

　　全面提升对内对外开放水平。深化区域合作交流，积极参与呼包银榆经济区和以呼包鄂为核心的自治区西部经济区建设。扩大向沿海发达地区开放，更大规模更高层次承接产业、资金、技术、人才转移。认真落实国家向北开放战略，加强与俄罗斯、蒙古国及欧洲的贸易往来，深化与国际友好城市的交流合作。推进出口加工区转型升级为综合保税区，打造外向型经济功能区。举办好呼和浩特投资贸易洽谈会、区域经济合作论坛，围绕首府优势产业及其配套延伸产业链，全面提升招商引资的规模和质量。今年力争引进国内资金640亿元以上，实际利用外资8.8亿美元以上。

　　着力提升科技创新能力和水平。高度重视科技创新在经济发展中的引领和驱动作用，围绕我市优势特色产业发展，增加科技创新资金投入，实施重大产业技术开发专项工程，努力掌握一批核心技术，培育一批具有自主知识产权的企业，提高产业核心竞争力。加强科技创新基地和平台建设，新认定国家高新技术企业7家，创建各类企业科技研发平台20家，新引进博士以上高层次科技创新创业人才20名。加快以留学人员创业园及科技孵化园为主的科技研发孵化基地建设，推进金山开发区升级为国家级高新技术产业开发区，支持鸿盛园区、金桥工业园区打造建设自治区级高新区，力争国家级云计算产业化基地通过认证。

　　(十)加强政府自身建设，努力建设人民满意的服务型政府

　　完成今年及今后一个时期的目标任务，回应和满足全市广大群众的新期待，各级政府责任重大。我们要进一步加强自身建设，全力打造为民、务实、高效、廉洁政府，全面提升政府服务水平。

　　大力转变工作作风。细化落实中央关于改进工作作风、密切联系群众的有关规定，切实精简会议、文件，减少和简化接待，清理和规范各种达标、评比、表彰以及论坛、庆典等活动，压缩“三公”经费。坚持崇尚实干、注重实效，在全市政府系统大兴“把业绩写在青城大地”的实干之风，推动各级政府工作人员把主要精力用在谋发展、促发展上，用在推进项目建设上，用在增进群众福祉上。继续大力推行机关马上办、主动办、上门办、公开办、灵活办的“五办”作风，下大力优化发展环境。各级领导干部要带头加强调查研究，带头密切联系群众，带头解决实际问题。

　　着力提高执行力和公信力。坚持决策的科学化、民主化，使各项政策更加符合实际、经得起检验。加强对政策执行情况的检查监督，切实做到令行禁止。强化行政问责，对失职渎职、不作为和乱作为的，要严肃追究责任。各地区、各部门对中央、自治区和我市的决策部署必须执行有力，绝不允许各自为政。各级行政机关及其公务员要自觉遵守宪法和法律，严格依法行政。切实改进行政执法工作，努力做到规范执法、公正执法、文明执法。支持人民法院和检察院依法独立行使审判权和监督权。

　　进一步加强廉政建设。继续完善惩治和预防腐败体系，推进廉政风险防控。认真贯彻执行《廉政准则》。加强和改进行政监察工作，认真治理政府工作人员以权谋私和渎职侵权问题，加大查处违法违纪案件工作力度。强化审计工作，加强对政府全口径预算决算的审查和监督。推进财政预算公开，让人民知道政府花了多少钱，办了什么事。加强对公务员的教育、管理和监督，努力建设一支政治强、业务精、作风好的公务员队伍。

　　各位代表，新的征程已经开启，新的目标催人奋进。让我们在党的十八大精神的指引下，在自治区党委、政府和市委的坚强领导下，抢抓机遇、锐意进取，凝心聚力、奋勇争先，为加快建设活力首府、美丽首府、和谐首府，全面实现“两个率先”目标而努力奋斗!
